# Preparation of Zinc Oxide with Core–Shell Structure and Its Application in Rubber Products

**DOI:** 10.3390/polym15102353

**Published:** 2023-05-18

**Authors:** Zhibin Wang, Zhanfeng Hou, Xianzhen Liu, Zhaolei Gu, Hui Li, Qi Chen

**Affiliations:** Key Laboratory of Rubber-Plastics, Ministry of Education/Shandong Provincial Key Laboratory of Rubber-Plastics, School of Polymer Science and Engineering, Qingdao University of Science and Technology, Qingdao 266042, China; 18742017071@163.com (Z.W.); 13012545139@163.com (Z.G.);

**Keywords:** core–shell structured zinc oxide, natural rubber, tread rubber, reduction in zinc oxide dosage

## Abstract

Zinc oxide is a crucial component in rubber products, but its excessive usage can lead to environmental damage. As a result, reducing the amount of zinc oxide in products has become a critical issue that many researchers aim to address. This study employs a wet precipitation method to prepare ZnO particles with different nucleoplasmic materials, resulting in ZnO with a core–shell structure. The prepared ZnO underwent XRD, SEM, and TEM analysis, indicating that some of the ZnO particles were loaded onto the nucleosomal materials. Specifically, ZnO with a silica core–shell structure demonstrated 11.9% higher tensile strength, 17.2% higher elongation at break, and 6.9% higher tear strength compared to the indirect method of ZnO preparation. The core–shell structure of ZnO also helps reduce its application in rubber products, thereby achieving the dual objective of protecting the environment and improving the economic efficiency of rubber products.

## 1. Introduction

Zinc oxide is an inorganic filler widely used in the rubber industry, and it has several functions. First, zinc oxide acts as an active agent in rubber to promote the vulcanization reaction, which helps to accelerate the rate of vulcanization and increase the degree of vulcanization, thus improving the physical and mechanical properties of rubber. Second, ZnO is used as a filler of rubber composites intended for products exhibiting increased heat conductivity. Furthermore, zinc oxide acts as an antioxidant that absorbs harmful ultraviolet rays, oxygen, and ozone to avoid their oxidation and the aging of rubber, thus prolonging the service life of rubber [1,2,3]. In addition, zinc oxide can be used as an antimicrobial agent to kill microorganisms by releasing oxygen and zinc ions, thus increasing the durability and service life of rubber products. In summary, zinc oxide is a very important rubber filler in rubber because of its multiple roles in promoting vulcanization, antioxidation, and antibacterial activity [4,5,6].

The rubber tire industry represents the largest consumer of zinc oxide, with approximately 50% of total zinc oxide usage being attributed to this industry [7]. Currently, micron-sized ZnO is used primarily as the curing active agent in the tire industry, and only a portion of the ZnO is involved in the activation of the curing reaction. As a result, a significant amount of ZnO remains in the rubber in the form of micron-sized particles, and residual Zn is released into the environment during tire operation, contributing to environmental pollution [8,9]. Despite the important role of ZnO in sulfur vulcanization, its concentration in rubber compounds, especially those used in aquatic environments, must be reduced to, at least, below 2.5 wt%, because zinc oxide is classified as being toxic to aquatic life. According to European Union Regulation (EC) No. 1272/2008 on classification, labeling, and packaging of substances and mixtures, ZnO was classified as Aquatic Acute 1 with hazard statement H400: Very toxic to aquatic life and Aquatic Chronic 1 with hazard statement H410: Very toxic to aquatic life with long lasting effects. The precaution recommended in this regulation is defined as P273: Avoid release to the environment. The release of zinc from rubber products occurs during their manufacture, use (dust created during the abrasion of tires on road surfaces), and recycling or disposal in landfills. A potential source of zinc in groundwater can also be rubber granulates made from end-of-life tires used to build artificial sports fields. Taking this into account, the problem of reducing the amount of zinc in rubber products is essential [10].

With advances in synthesis technology, various methods have been employed to prepare ZnO nanoparticles as a substitute for conventional ZnO. The methods and conditions for preparing ZnO nanoparticles have been extensively investigated and can be broadly categorized as solid-phase, liquid-phase, and gas-phase methods based on the phase state of the reactants [11,12,13,14]. It has been demonstrated that decreasing the particle size of ZnO nanoparticles results in a rougher surface and uneven atomic steps, leading to increased contact surface and chemical activity [15,16,17,18]. In contrast, the particle size of ZnO nanoparticles is considerably smaller than that of ZnO, making it theoretically feasible to replace ZnO and reduce the amount of zinc used [19,20,21]. The use of high-specific-surface-area ZnO nanoparticles can increase the contact area between ZnO and rubber, improve the efficiency of ZnO in the activation reaction (by increasing the reaction rate and reducing energy consumption during vulcanization), and simultaneously reduce the amount of ZnO used without compromising the enhancement effect [22,23,24,25]. According to thermodynamic theory, smaller particle sizes result in reduced dispersion effectiveness. ZnO nanoparticles tend to agglomerate due to their small size and high specific surface energy, thus limiting their nano-effect [26,27,28]. To address this issue, researchers have employed strategies such as loading ZnO nanoparticles or preparing core–shell structured particles. For instance, Magdalena G. et al. [6] used a gel method to coat ZnO nanoparticles onto the surface of SiO_2_ to investigate the effect of SiO_2_@ZnO core–shell structured nanoparticles on the kinetics of carboxylated nitrile rubber. Yalan L. et al. [29] used a wet blending method to load ZnO onto the surface of cellulose fibers to study the dispersion of cellulose fibers in the rubber matrix and its impact on the mechanical strength of natural rubber. Furthermore, Zeinab D.G. et al. [30] prepared CoO.CaO/ZnO core–shell structured particles and examined their effect on the mechanical properties of nitrile butadiene rubber (NBR), with ZnO as the core and CoO and CaO as the shell. This approach improved the tensile strength of NBR and enhanced the compatibility between ZnO and NBR. In addition, some researchers have employed nanoscale active ZnO with a micron-level carrier coating structure as the starting material and added various low-molecular-weight PIBs as dispersion aids to the nano-active ZnO powder to achieve surface modification, thereby improving several surface properties such as agglomeration adsorption and dispersion. This strategy ultimately enhances the compatibility of nano-active ZnO with rubber materials.

In this study, core–shell structured zinc oxide nanoparticles were synthesized via the wet precipitation method, using various materials including calcium carbonate, barium sulfate, silicon dioxide, thermally cracked carbon black, and graphene as the core material. The resulting core–shell structured zinc oxide nanoparticles were characterized in terms of morphology, particle size, and the extent of zinc oxide loading onto the core material. Subsequently, the prepared zinc oxide nanoparticles were incorporated into the formulation of semi-steel radial tire tread rubber, and the effects on the vulcanization characteristics, mechanical properties, friction properties, aging properties, and dynamic thermomechanical properties of the rubber were studied.

## 2. Experimental Part

### 2.1. Experimental Materials

Zinc chloride used in the preparation was sourced from Shandong Xuanhai Chemical Co., Ltd. (Heze, China), whereas sodium carbonate was obtained from Tianjin Jinhui Pharmaceutical Group Co., Ltd. (Tianjin, China) The calcium carbonate used in the experiment was provided by Changzhou Calcium Carbonate Co., Ltd. (Changzhou, China) For the preparation of core–shell structure zinc oxide of barium sulfate, Shandong Qiyi Chemical Technology Co., Ltd. (Weifang, China) provided the barium sulfate, and Shandong Bluestar Dongda Chemical Co., Ltd. (Zibo, China) provided the silicon dioxide for the core–shell structure zinc oxide of silica. The graphene used in the experiment was sourced from Chinese Academy of Sciences Chengdu Organic Chemistry Co., Ltd. (Chengdu, China), whereas the thermal cracking carbon black was obtained from Jiangxi Black Cat Carbon Black Co., Ltd. (Jingdezhen, China). The indirect method of zinc oxide used in the experiment was provided by Anqiu Hengshan Zinc Industry Co., Ltd. (Anqiu, China), whereas the other complexes were provided by Guangzhou Chemical Reagent Factory. To prepare the zinc oxide with core–shell structure, the aforementioned raw materials were used, and the experiment was carried out according to the previously described method.

### 2.2. Preparation of Zinc Oxide with Core–Shell Structures

**Figure 1:**
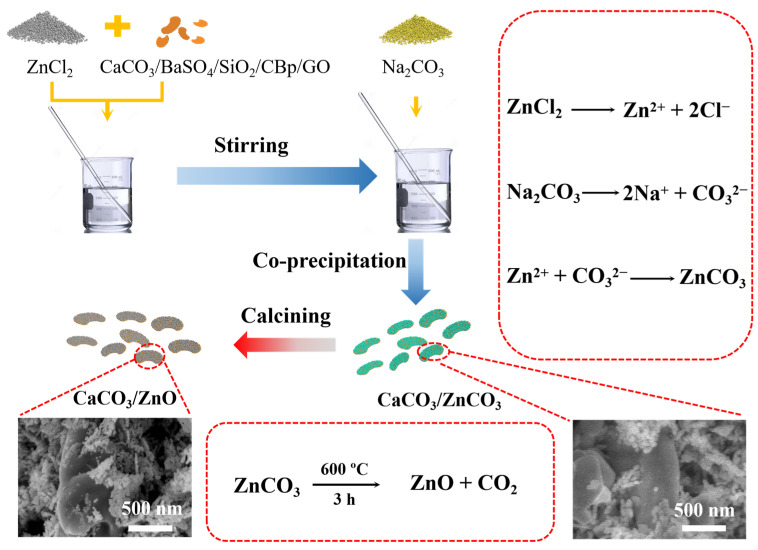
Preparation process of core–shell structure zinc oxide.

To prepare core–shell structured ZnO particles, analytical grade zinc chloride, sodium, carbonate calcium carbonate (CaCO_3_), barium sulfate (BaSO_4_), silicon dioxide (SiO_2_), pyrolysis of carbon black (CBp), grapheme oxide (GO), and distilled water were used. To prepare ZnO@CaCO_3_, 138 g of zinc chloride was dissolved in 330 mL of distilled water. Alternatively, 108 g of sodium carbonate was added to 500 mL of distilled water. The prepared zinc chloride solution was added to the beaker with 81 g of calcium carbonate solids and stirred at 80 °C to make the zinc chloride solution infiltrate the surface of the calcium carbonate solids. Then, the prepared sodium carbonate solution was slowly added to beaker and the solution was continuously stirred at 80 °C in an oil bath. A viscous and honey-like gel was obtained after continuously stirring the solution for 30 min at 80 °C. The solution was washed with water three times and dried for 12 h. Lastly, for the calcination process, a small amount of provided powers were put in an alumina crucible before being placed into a 600 °C furnace. The heating rate was 5 °C/min, and the heating operation was 3 h [31]. Figure 1 presents the process of core–shell structured ZnO. The core–shell structured zinc oxide/barium sulfate, zinc oxide/silica, zinc oxide/pyrolysis of carbon black, and zinc oxide/graphene were prepared by replacing the core–shell materials in the same way.

### 2.3. Preparation of Natural Rubber-Based Nanocomposites

The rubber and auxiliary were mixed in the compactor according to the formulation shown in Table 1. The total mixing time is 8 min and then vulcanized at 143 °C to obtain the best cure time (t_90_). The rubber compounds obtained by adding different types of zinc oxide were named R-ZnO, R-ZnO@CaCO_3_, R-ZnO@BaSO_4_, R-ZnO@SiO_2_, R-ZnO@CBp, and R-ZnO@GO.

### 2.4. Characterization

The crystal structure of the core–shell structured powdered ZnO was determined using a D-MAX2500/PC X-ray diffractometer (Nippon Rigaku Co., Ltd., Tokyo, Japan) with a test range of 10° to 80° and a scanning rate of 5°/min. The morphological characteristics were observed using a scanning electron microscope (JSM-7500F; Nippon Electron Co., Ltd., Tokyo, Japan) with an acceleration voltage of 3 kV. The transmission electron microscope (JEOL-JEM-2100; Japan Electron Co., Ltd., Tokyo, Japan) was used to investigate the microscopic morphology of ZnO with a core–shell structure made from different nucleosomal materials. To test the properties of the prepared core–shell structured ZnO, it was added to a natural rubber formulation. All vulcanization properties were measured using a vulcanometer (MDR2000, Alpha Technologies, Hudson, Ohio, USA) at 143 °C. The mechanical properties were tested using an electronic tensile machine (I-7000S, High Iron Co., Ltd., Taipei, Taiwan) with the sample strips cut into dumbbell-shaped strips with a length of 75 mm, thickness of 2.00 ± 0.03 mm, and working width of 4 mm. The tensile properties were measured at a speed of 500 mm/min. Tear strength was measured using the right-angle tear mode C according to ASTM D624. The thermal oxygen aging chamber was used to age the cut tensile and tear sample strips at 100 °C for 72 h. Abrasion was tested using a DIN abrasion machine (GT-7012-D, GOTECH Co., Ltd., Taichung, Taiwan) with a pressure of 10 N and a roller speed of 40 r/min. The dynamic mechanical analysis was performed using a dynamic thermomechanical analyzer (242, NETZSCH, Selb, Germany) with a test temperature range from −60 °C to 80 °C at a frequency of 3 Hz and a ramp rate of 3 °C/min.

## 3. Results and Discussion

### 3.1. Characterization of Core–Shell Structure ZnO

#### 3.1.1. XRD

**Figure 2:**
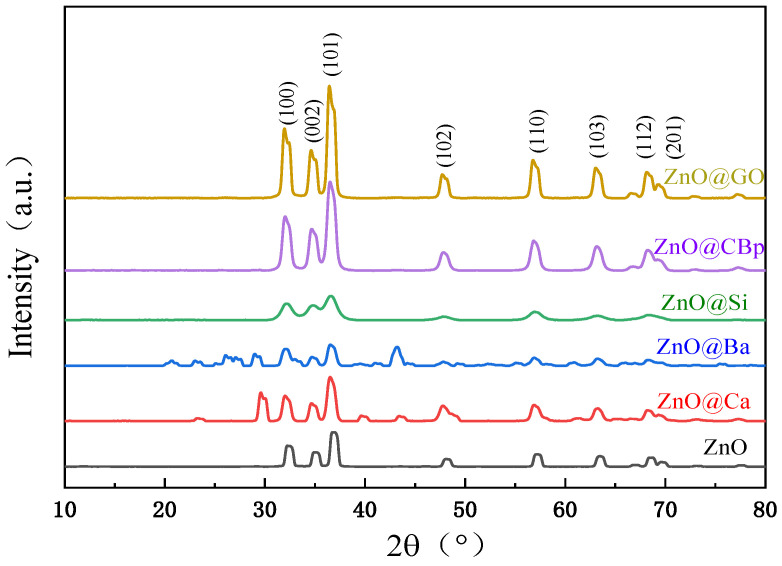
XRD of zinc oxide with core–shell structure of different nuclear materials.

The crystallinity patterns and structures of the synthesized samples were analyzed using X-ray diffraction (XRD). Figure 2 shows the XRD patterns of commercially available ZnO composites prepared by the indirect method, which exhibited diffraction peaks at 31.6°, 34.4°, 36.1°, 47.3°, 56.3°, 62.6°, and 67.6° corresponding to (100), (002), (101), (102), (110), (103), and (112) planes. The core–shell structured ZnO prepared with nucleosomal materials such as graphene and pyrolysis of carbon black showed similar peak patterns, indicating that the structure of ZnO on the surface of CBp and GO is primarily in the hexagonal phase, which was produced at 600 °C. Hence, for the core–shell structured ZnO prepared with nucleosomal materials such as SiO_2_, CaCO_3_, and BaSO_4_, some different diffraction peaks were detected in the patterns. The XRD patterns of Ca and Ba exhibited characteristic diffraction peaks of calcium carbonate at 23.0°, 29.4°, and 39.3°, and of barium sulfate at 22.7°, 24.8°, 26.8°, and 31.5°, respectively. These observations suggest that the zinc oxide was attached to the surface of calcium carbonate and barium sulfate.

#### 3.1.2. SEM

**Figure 3:**
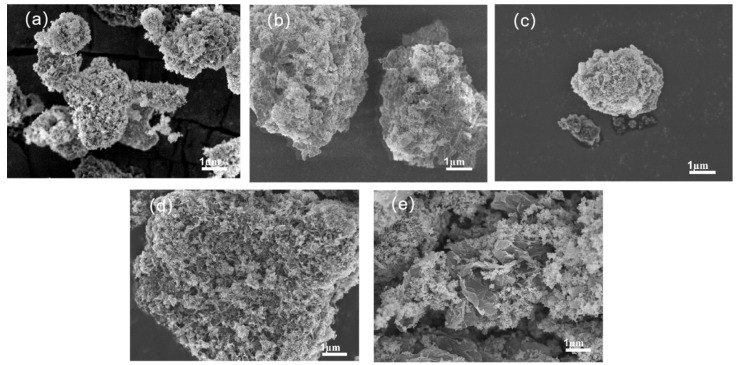
SEM image of the core–shell structure zinc oxide: (**a**) CaCO_3_; (**b**) BaSO_4_; (**c**) SiO_2_; (**d**) CBp; (**e**) GO.

The morphology of ZnO with different nucleosomal materials is presented in Figure 3. The high-resolution images of ZnO@CaCO_3_ powder showed the formation of ZnO nanoparticles, which were spherical in size, uniform, and dense. ZnO was well coated around the nucleosomal material in Figure 3a. In Figure 3b, ZnO mainly adhered to the carrier surface in an irregular cylindrical shape, with particles adhering to each other in agglomeration. In contrast, the flaky structure of zinc oxide shown in Figure 3c,d had numerous bumpy blocks, indicating poor dispersion and slight agglomeration of zinc oxide monomers distributed on the carrier surface in a sea-urchin-like crystal form. As displayed in Figure 3e, most GO nanosheets are stacked, curled, and entangled together. The synthesized nano-ZnO exhibits an obvious tendency for the nanoparticles to agglomerate. It should be noted that the surface of GO nanosheets is covered by densely packed and irregularly shaped nano-ZnO on a large scale. Some nano-ZnO particles that are grown on the brink of the interlayer and inside the interlayer of GE nanosheets did not wrap the nucleosomal material well, and most of the graphene material was exposed.

#### 3.1.3. TEM

**Figure 4:**
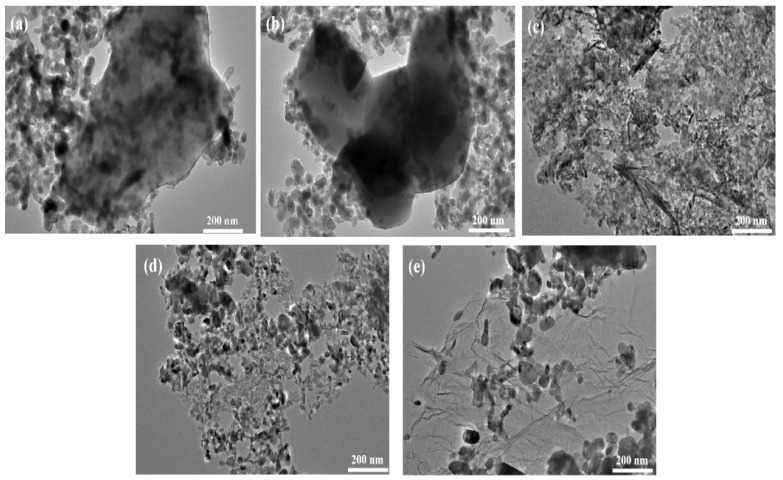
TEM image of the core–shell structure zinc oxide: (**a**) CaCO_3_; (**b**) BaSO_4_; (**c**) SiO_2_; (**d**) CBp; (**e**) graphene.

TEM was performed to investigate the ZnO with core–shell structure, as shown in Figure 4. Figure 4a shows that a number of the ZnO particles were electrostatically adsorbed onto the surface of calcium carbonate solid, whereas others were free and not adsorbed. In Figure 4b, the size of the ZnO particles was between 60 and 70 nm. In Figure 4c, only a small portion of the needle-like ZnO structure was attached to the SiO_2_ surface, whereas the ZnO morphology on the silica nucleosome material was needle-like. Figure 4d illustrates that the ZnO particles were attached to the nucleosome material in a spherical structure, and it is clear that the ZnO particles were agglomerated together. Figure 4e shows the GO nanosheets are decorated by nano-ZnO 60 nm in diameter. Notably, some nano-ZnO particles are dispersed on the surface of the wrinkled GO nanosheets, and some are covered or wrapped by thin GO nanosheets, in agreement with the SEM observations.

### 3.2. Effect of Core–Shell ZnO of Different Core Materials on the Vulcanization Performance of Tire Tread Rubber

The vulcanization performance of ZnO with different nucleosomal materials in tire tread rubber was evaluated, as shown in Table 2. The results indicate that ZnO with a nucleosomal structure exhibits slightly lower M_H_-M_L_ values than indirect ZnO, which may be due to its lower percentage among the same amount of ZnO, resulting in a slightly lower crosslink density. The core–shell structured zinc oxide exhibits a shorter positive vulcanization time and the fastest vulcanization rate, with a shorter scorch time compared to indirect zinc oxide. This can be attributed to the small particle size, large specific surface area, and severe lack of coordination of the core–shell structured ZnO compared to conventional ZnO, resulting in higher reactivity. In contrast, the vulcanization rate of R-ZnO@CBp and R-ZnO@GO core materials was slower due to the thermal cracking of carbon black and the easy agglomeration of graphene in the rubber, resulting in insufficient reaction between ZnO and the vulcanizing agent.

### 3.3. Effect of Core–Shell ZnO of Different Core Materials on the Mechanical Properties of Tire Tread Rubber

**Figure 5:**
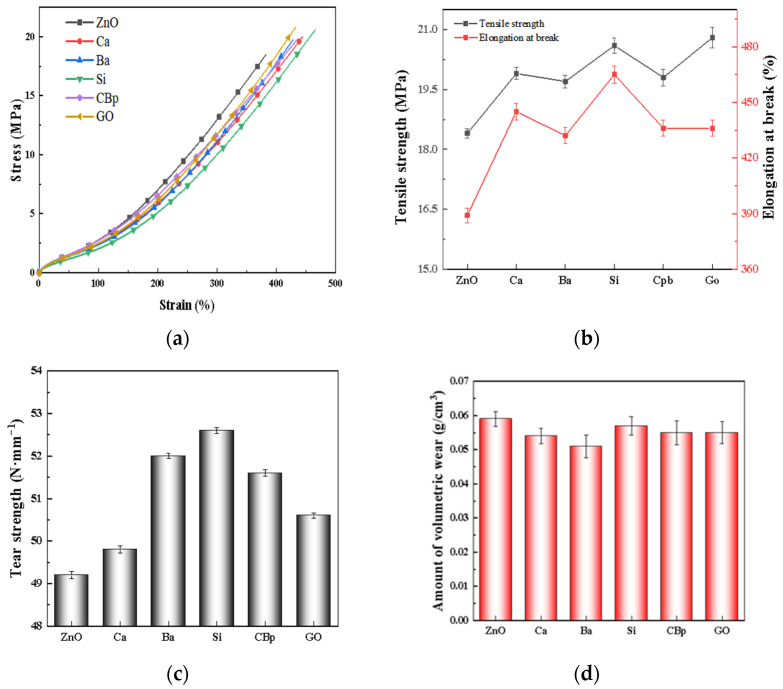
Mechanical properties of zinc oxide with core–shell structure. (**a**) Stress-strain curve; (**b**) tensile strength, elongation at break; (**c**) tear strength; (**d**) wear resistance.

The mechanical properties of the rubber compounds are shown in Table 3 and Figure 5. It shows that the tensile strength and elongation at break of the core–shell structured ZnO specimens are greater than those of R-ZnO, probably because of the large specific surface area, better dispersion, and greater crosslinking of the core–shell structured ZnO, which exhibits excellent mechanical properties. For core–shell structured ZnO, R-ZnO@Si has better performance, which may be due to the small size and good dispersion of ZnO attached to silica, which can be effectively combined with the promoter. On the other hand, silica acts as a reinforcing system indirectly to increase the performance of the adhesive. Comparing the tearing properties, the core–shell structured ZnO exhibits generally higher tear strength than the indirect method ZnO. The high surface activity of the small particle size zinc oxide in the core–shell structured zinc oxide promotes a dense mesh structure that increases the degree of crosslinking of the rubber and limits the movement of the molecular chains. As a result, the elastic modulus in the directional direction is smaller than that in the vertical direction, hindering the crack expansion.

As shown in Figure 5d, R-ZnO has the highest wear resistance, which is due to the lower crosslink density and higher wear rate, which is due to the lower degree of crosslinking and susceptibility to damage by mechanical stress. Due to the higher crosslink density of R-ZnO, the number of crosslinking sites per unit volume is higher, and the number of effective molecular chains carrying mechanical stress is higher compared to that of ZnO with a core–shell structure, resulting in a higher wear resistance. Similarly, for ZnO with a core–shell structure, the high degree of crosslinking leads to excellent wear resistance.

### 3.4. Effect of Core–Shell ZnO of Different Core Materials on the Dynamic Mechanical Properties of Tire Tread Rubber

**Figure 6:**
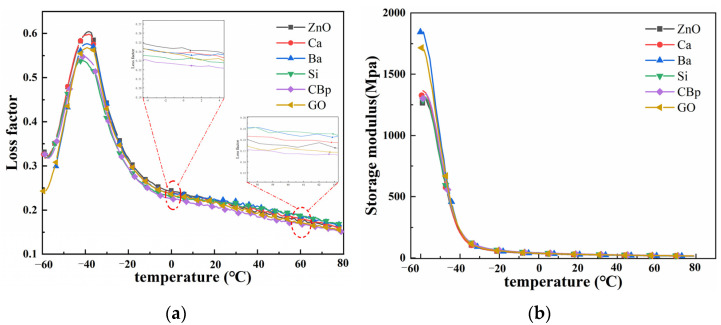
DMA of core–shell ZnO with different core materials. (**a**) tanδ; (**b**) storage modulus.

The loss factors of the materials are shown in Figure 6a, and there is a significant decrease in the loss factor peak of the core–shell structured ZnO compared to the indirect method ZnO, which indicates that the core–shell structure reduces the motion of the molecular chains of the composites. tanδ at 0 °C and 60 °C then characterizes the wet-slip resistance and rolling resistance of the rubber material for tires. The loss factor curve at 0 °C shows that the wet slip resistance of the composites with the addition of ZnO with core–shell structure decreases. It indicates that the indirect method zinc oxide has stronger interaction with the matrix and higher energy loss from the movement of molecular chains. In contrast, at 60 °C, the adhesive with thermally cracked carbon black and graphene as core–shell materials has a better rolling resistance performance, probably because the core–shell structured ZnO promotes the dispersion of activator after loading, enhances the vulcanization, and builds a stronger spatial crosslinking network, which makes the hysteresis loss inside the material lower, resulting in less internal friction and lower rolling resistance. Compared with the indirect ZnO adhesive, the energy storage modulus of the adhesive with barium sulfate nucleosome material increased by 42.5%, whereas the energy storage modulus of the adhesive with graphene as the nucleosome material increased by 32.5%.

### 3.5. Effects of Core and Shell ZnO with Different Core Materials on the Aging Properties of Tire Tread Compounds

**Figure 7:**
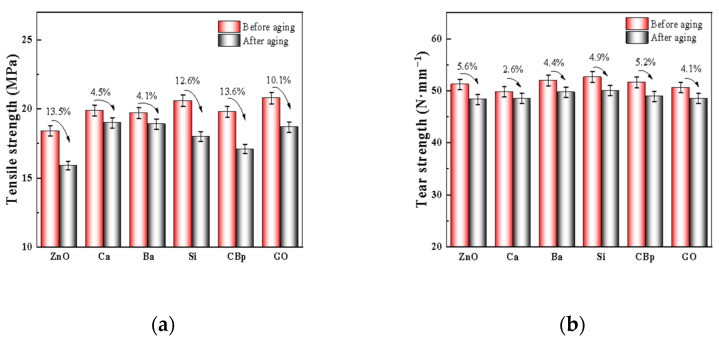
Effect of core–shell zinc oxide with different core materials on the aging performance of tire tread rubber. (**a**) Tensile strength after aging; (**b**) tear strength after aging.

The effects of different core–shell materials of ZnO on the aging performance of rubber are shown in Figure 7. The performance of indirect method ZnO decreases more after aging; the possible reason is that the reaction between indirect method ZnO and accelerator generates less active zinc salts, which leads to low utilization of the vulcanizing agent, and the crosslinked network is dominated by polysulfide bonds, which are less thermally stable, so the performance decreases more obviously after thermal-oxidative aging. In comparison with the core–shell structure zinc oxide of different nucleosomal materials, R-ZnO@Ca and R-ZnO@Ba have better aging resistance, and the rest of the core–shell structure zinc oxide has poorer aging resistance, which may be attributed to the fact that calcium carbonate and barium sulfate core–shell structure zinc oxide in rubber are not easily agglomerated to trigger the unreacted vulcanizing agent, resulting in the crosslinking of unreacted double bonds on the rubber molecular chain; thus, the performance degradation is to a lesser extent. The difficulty of dispersion of thermally cracked carbon black and graphene in rubber makes the content of the unreacted vulcanizing agent decrease, which makes it difficult to trigger the crosslinking reaction of the double bonds on the rubber molecular chain again; i.e., the ability to resist external damage is reduced, so the degradation of rubber performance is more obvious.

## 4. Conclusions

This paper proves the core–shell structured zinc oxide was synthesized by a simple wet precipitation method and post-processing approaches without using harmful chemicals. The XRD results showed that the intense and sharp peaks in the ZnO hexagonal were highly crystalline. The SEM and TEM analysis revealed that ZnO nanoparticles (nano-ZnO) are successfully anchored onto carbonate calcium carbonate (CaCO3), barium sulfate (BaSO4), silicon dioxide (SiO2), pyrolysis of carbon black (CBp), and graphene oxide (GO) sheets. The performance of NR/SBR/BR compounds was studied by adding ZnO with different nucleosomes to the formulation of semi-steel radial tire tread rubber. The results showed that the core–shell structured ZnO with low ZnO content possesses a higher vulcanization efficiency and much stronger reinforcement effect on the mechanical performance properties of NR/SBR/BR compounds compared with the indirect method zinc oxide, results of which are positively correlated with the good dispersion of ZnO throughout the NR matrix, the enhanced interfacial interaction between the ZnO and the matrix, and the high vulcanization efficiency of nano-ZnO. For the different core–shell structured zinc oxide materials, the ZnO@silica-based rubber is superior in mechanical and abrasion resistance, and the calcium-carbonate-based core–shell structured zinc oxide has excellent aging resistance. Overall, the performance of core–shell structured zinc oxide product is basically the same as that of indirect zinc oxide products, while the amount of zinc oxide in rubber can be reduced, which can also lead to a reduction in the production cost of rubber products. Accordingly, the core–shell structured ZnO with lower content is very competitive for preparing rubber composites with high performance, and it may be regarded as a substitute of conventional ZnO for application in rubber composites.

## Figures and Tables

**Table 1:** Tire tread rubber experimental formula (phr).

	R-ZnO	R-ZnO@Ca	R-ZnO@Ba	R-ZnO@Si	R-ZnO@CBp	R-ZnO@GO
BR9000	20	20	20	20	20	20
SBR	89	89	89	89	89	89
NR	15	15	15	15	15	15
Silica	65	65	65	65	65	65
N375	20	20	20	20	20	20
Indirect method ZnO	3	-	-	-	-	-
ZnO@CaCO_3_	-	3	-	-	-	-
ZnO@BaSO_4_	-	-	3	-	-	-
ZnO@SiO_2_	-	-	-	3	-	-
ZnO@CBp	-	-	-	-	3	-
ZnO@Go	-	-	-	-	-	3
Si69	9.5	9.5	9.5	9.5	9.5	9.5
SA	2	2	2	2	2	2
S	1.3	1.3	1.3	1.3	1.3	1.3
CZ	1.8	1.8	1.8	1.8	1.8	1.8

**Table 2:** Effect of core–shell zinc oxide with different core materials on vulcanization performance of tire tread rubber.

	R-ZnO	R-ZnO@Ca	R-ZnO@Ba	R-ZnO@Si	R-ZnO@CBp	R-ZnO@GO
**M_L_/dN·m**	1.32	1.45	1.70	1.88	1.71	1.65
**M_H_/dN·m**	18.56	17.80	18.68	18.99	18.62	18.55
**T_10_/s**	444	372	329	319	437	446
**T_90_/s**	1997	1526	1405	1343	1809	1878
**M_H_-M_L_/dN·m**	17.24	16.35	16.98	17.11	16.91	16.90

**Table 3:** Mechanical properties of zinc oxide with core–shell structure.

	R-ZnO	R-ZnO@Ca	R-ZnO@Ba	R-ZnO@Si	R-ZnO@CBp	R-ZnO@GO
**Tensile strength/MPa**	18.4	19.9	19.7	20.6	19.8	20.8
**Elongation at break/%**	389	445	432	465	436	436
**Modulus at 100% strain/MPa**	3.5	3.5	3.4	3.45	3.6	3.8
**Modulus at 300% strain/MPa**	12.6	13.2	12.8	13.0	13.7	14.0
**Tear strength/N ·mm^−1^**	49.2	49.8	52.0	52.6	51.6	50.6
**Hardness**	75	75	75	76	77	76

## Data Availability

The data that support the findings of this study are available from the corresponding author upon reasonable request.
